# Chronic toxicity of amitraz, coumaphos and fluvalinate to *Apis mellifera* L. larvae reared *in vitro*

**DOI:** 10.1038/s41598-018-24045-3

**Published:** 2018-04-04

**Authors:** Pingli Dai, Cameron J. Jack, Ashley N. Mortensen, Tomas A. Bustamante, James D. Ellis

**Affiliations:** 1 Key Laboratory of Pollinating Insect Biology, Institute of Apicultural Research, Chinese Academy of Agricultural Sciences, Beijing, 100093 China; 2 Honey Bee Research and Extension Laboratory, Entomology and Nematology Department, University of Florida, Gainesville, Florida 32611 USA

## Abstract

The effects of chronic exposure to common acaricides on *Apis mellifera* survival, developmental rate and larval weight were tested in the laboratory. Larvae were reared *in vitro* and fed a diet containing amitraz: 1.5, 11, 25 and 46 mg/L; coumaphos: 1.8, 6, 8 and 25 mg/L; or fluvalinate: 0.1, 1, 2.4 and 6 mg/L. The dependent variables were compared for groups feeding on treated diets and control diets: positive control, 45 mg/L dimethoate; solvent control; and negative control. Bee survival decreased in the 46 mg/L amitraz and 25 mg/L coumaphos treatments but not in any fluvalinate treatment. Furthermore, the developmental rate decreased in individuals treated with 46 mg/L amitraz. In our study, larvae exposed to acaricides at concentrations similar to maximum residue in pollen and honey/nectar had no detectable change in survival or developmental rate. Given that pollen and honey/nectar represent only a small part of larval diet, we suggest that residues of amitraz, coumaphos and fluvalinate at the levels we tested are unlikely to impact immature worker bee survival in the field, though our data do not preclude any sublethal effects that may result from bee exposure to these compounds or possible synergisms when they co-occur in bee colonies.

## Introduction

The parasitic mite *Varroa destructor* is one of the most devastating pests of the western honey bee (*Apis mellifera* sspp. L.)^1,2^, and is considered to play a central role in honey bee population decline^3^. Pesticides also can impact colony health negatively^4,5^. Generally, the highest pesticide residues found in honey bee colonies are chemicals that have been introduced by beekeepers into colonies intentionally in an attempt to mitigate the effects of *Varroa* infestations^6,7^. Beekeepers employ a range of synthetic acaricides such as amitraz (formamidine), coumaphos (organophosphate) and fluvalinate (pyrethroid) to control *Varroa* populations^6^.

Amitraz, coumaphos, and fluvalinate are approved for use in honey bee colonies and, when used according the label, do not impact the bees acutely. However, very low doses/concentrations of these acaricides may sublethally affect bee physiology, neurology, metabolism, and/or behavior^4,8^. Moreover, chemicals accumulate in the honey, wax, and pollen within the beehive and this may lead to chronic exposure of adult and immature bees to sublethal doses of acaricides^9–11^. This, in turn, can be detrimental to the fate of the colony because sublethal effects, such as altered physiology, impaired function, or reduced lifespan of the adult bees within the hive, can result in rapid depopulation of a colony^12^.

Sublethal effects of amitraz, coumaphos, and fluvalinate have been demonstrated for adult honey bees. Amitraz can lead to behavioral changes in adult honey bees^13^ while fluvalinate may impact queen performance^14^. Furthermore, dermal applications of amitraz (1.25 μg/bee) and fluvalinate (1.25 μg/bee) significantly impact honey bee mortality, but these concentrations exceed those to which honey bees would normally be exposed^15^. Repeated applications of coumaphos to hives leads to elevated concentrations of coumaphos in beeswax^7,13^. The presence of coumaphos and fluvalinate in beeswax can decrease brood survival^14^, and the simultaneous application of coumaphos and fluvalinate can increase bee mortality^16,17^ and decrease three-day brood survival^18^. Drones exposed to coumaphos or fluvalinate have been shown to have reduced sperm viability^9^, and drones exposed to fluvalinate during immature development experience increased mortality and reduced body weight and tend toward lower sperm counts^19^. The application of coumaphos to colonies can impact queen development and negatively impact queen health^20^ and residues in wax queen cells can reduce developing queen survival and weight. Ultimately, acaricides can alter physiological functions, immune responses, and detoxification functions in the exposed bees, possibly rendering them more susceptible to pathogens and pesticides^21^.

Most investigations into pesticide impacts on honey bees are focused on adult bees, even though brood (immature bees: eggs, larvae and pupae) is crucial to colony fitness. A robust risk assessment for any pesticide should include an evaluation of possible sublethal effects on honey bee brood^22,23^. It is prudent that we determine the potential impacts of amitraz, coumaphos, and fluvalinate on immature honey bees given their widespread use in honey bees hives to control *Varroa*.

Unfortunately, experiments conducted within a honey bee hive can be biased by many uncontrollable factors such as colony strength, weather conditions, food availability, and preexisting pesticide residues^7,24^. Much of this bias can be overcome by rearing honey bee larvae and exposing them to toxins in the laboratory. An *in vitro* methodology has been developed for rearing bee larvae^25,26^. This technique can be used to determine pesticide toxicity to larvae^25–27^. In this study, we evaluated survival, developmental rate and larval weight of *in vitro*-reared honey bees exposed chronically to varying concentrations of amitraz, coumaphos, or fluvalinate as larvae.

## Results

### Survival

Control average total survival was 88.3% (negative control), 85.4% (solvent control: 0.5% acetone), 81.7% (solvent control: 0.5% methanol), and 11.7% (positive control: 45 mg/L dimethoate), thus validating the test design.

**Figure 1:**
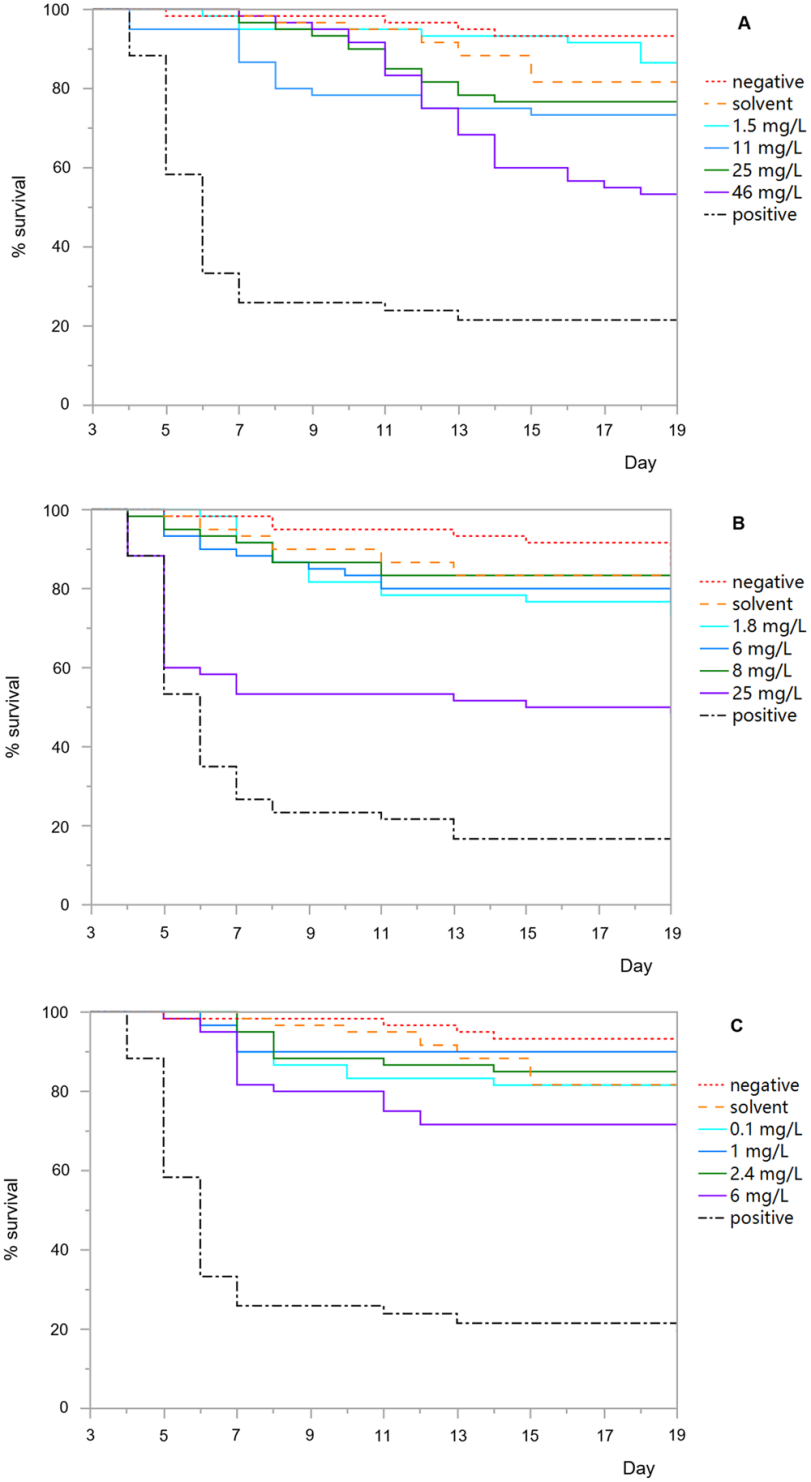
Total survival of honey bees exposed to concentrations of (**A**) amitraz, (**B**) coumaphos or (**C**) fluvalinate during larval development on D3 thru D6 after grafting (n = 60 larvae per test substance). Larvae were fed a dimethoate-contaminated diet (45 mg/L) as a positive control, acetone- or methanol-contaminated diet as a solvent control, and no contaminated diet as a negative control.

Total survival for larvae fed 1.5 mg/L amitraz and larvae fed the negative control (χ^2^ = 1.38, *p* = 0.24, Table S1, Fig. 1A) or solvent control diets (χ^2^ = 0.54, *p* = 0.46, Table S1, Fig. 1A) were not significantly different from one another. Additionally, there were no statistical differences between total survival of larvae fed 11 mg/L (χ^2^ = 2.20, *p* = 0.14) or 25 mg/L (χ^2^ = 1.82, *p* = 0.18) amitraz and that of larvae fed the solvent control diet (Table S1, Fig. 1A). However, there was a statistically significant difference between total survival of larvae fed 11 mg/L (χ^2^ = 9.83, *p* = 0.002) or 25 mg/L (χ^2^ = 9.52, *p* = 0.002) of amitraz and that of larvae fed the negative control diet (Table S1, Fig. 1A). Furthermore, there was a statistically significant decrease in the total survival of larvae fed 46 mg/L amitraz compared to those fed the negative control or the solvent control diets (all *p* < 0.0001, Table S1, Fig. 1A).

There were not statistically detectable differences between the total survival of larvae fed 1.8, 6 or 8 mg/L coumaphos and those fed negative control or solvent control diets (all *p* > 0.05, Table S2, Fig. 1B). Conversely, larvae that were fed the highest does of coumaphos (25 mg/L) had significantly lower total survival than did those fed the negative and solvent control diets (all *p* < 0.0001, Table S2, Fig. 1B).

There were no significant differences between total survival rates of larvae fed a diet with any concentration of fluvalinate (0.1, 1, or 2.4 mg/L) and those fed negative control or solvent control diet (all *p* > 0.05, Table S3, Fig. 1C). There was a statistically significant decrease in the survival rate of larvae fed 6 mg/L fluvalinate compared to those fed the negative control diet (χ^2^ = 11.00, *p* = 0.001), but no significant difference between 6 mg/L fluvalinate and solvent control (χ^2^ = 2.90, *p* = 0.09, Fig. 1C).

**Figure 2:**
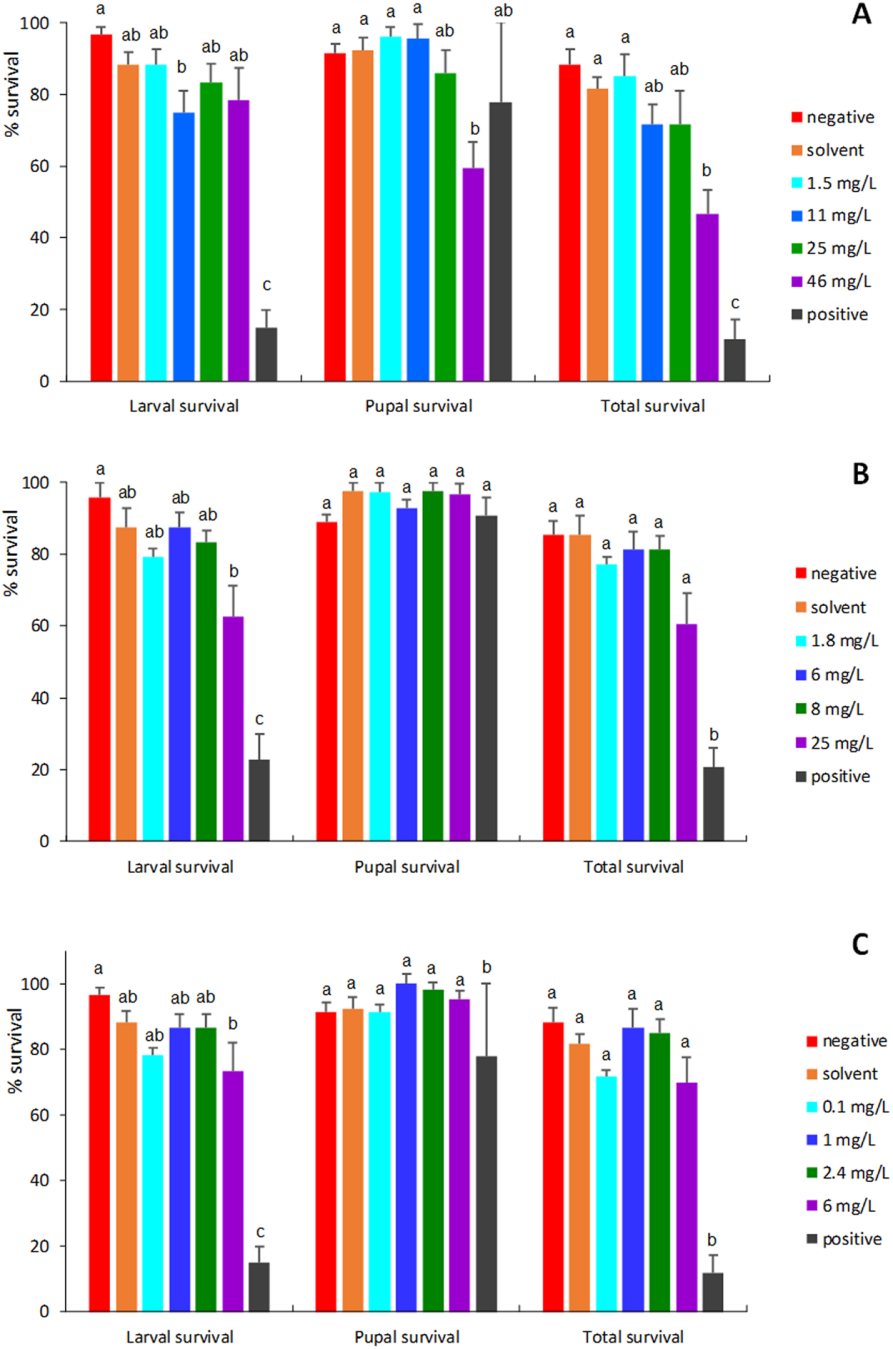
Larval survival, pupal survival, and total survival (mean % survival ± SE) of honey bees reared *in vitro* and exposed to (**A**) amitraz, (**B**) coumaphos and (**C**) fluvalinate in their diet at D3, D4, D5 and D6 after grafting. Larvae were fed a dimethoate-contaminated diet (45 mg/L) as a positive control, acetone- or methanol-contaminated diet as a solvent control, or no contaminated diet as a negative control. Bars with the same letter within survival type (larval, pupal, and total) are not different at α ≤ 0.05.

A one-way ANOVA indicated differences between treatment groups in the amitraz study for larval survival (F_34_ = 24.09, *p* < 0.0001), pupal survival (F_34_ = 3.26, *p = *0.0147), and total survival (F_34_ = 19.23, *p* < 0.0001). Larval survival in individuals fed diet with 11 mg/L amitraz was significantly lower than that of larvae feeding on the negative control diet, though not the solvent control diet, or the other concentrations of amitraz (Fig. 2A1). Pupal survival for individuals fed a diet with 46 mg/L of amitraz was significantly lower than that for individuals fed the negative and solvent control diets, and the diet with 1.5 mg/L amitraz (Fig. 2A2). Total survival was unaffected in individuals fed 1.5, 11 and 25 mg/L amitraz (Fig. 2A3) though it was significantly lower than that of the negative and solvent controls for individuals fed 46 mg/L amitraz (Fig. 2A3).

A one-way ANOVA indicated differences between treatment groups in the coumaphos study for larval survival (F_34_ = 20.08, *p* < 0.0001) and total survival (F_34_ = 22.02, *p* < 0.0001), but not pupal survival (F_34_ = 1.15, *p = *0.3711). Though significant differences between groups existed for larval and pupal survival, no meaningful patterns were discernable (Fig. 2B).

A one-way ANOVA indicated differences between treatment groups in the fluvalinate study for larval survival (F_34_ = 26.84, *p* < 0.0001), pupal survival (F_34_ = 3.58, *p = *0.0093), and total survival (F_34_ = 24.54, *p* < 0.0001). Though significant differences between groups existed for larval, pupal and total survival, no meaningful patterns were discernable (Fig. 2C).

Larval and total survival were always significantly lower for individuals fed the positive control diet than for ones fed any other diet for all three compounds (P < 0.05, Fig. 2). This effect was not seen for pupal survival.

### Developmental Rate

**Figure 3:**
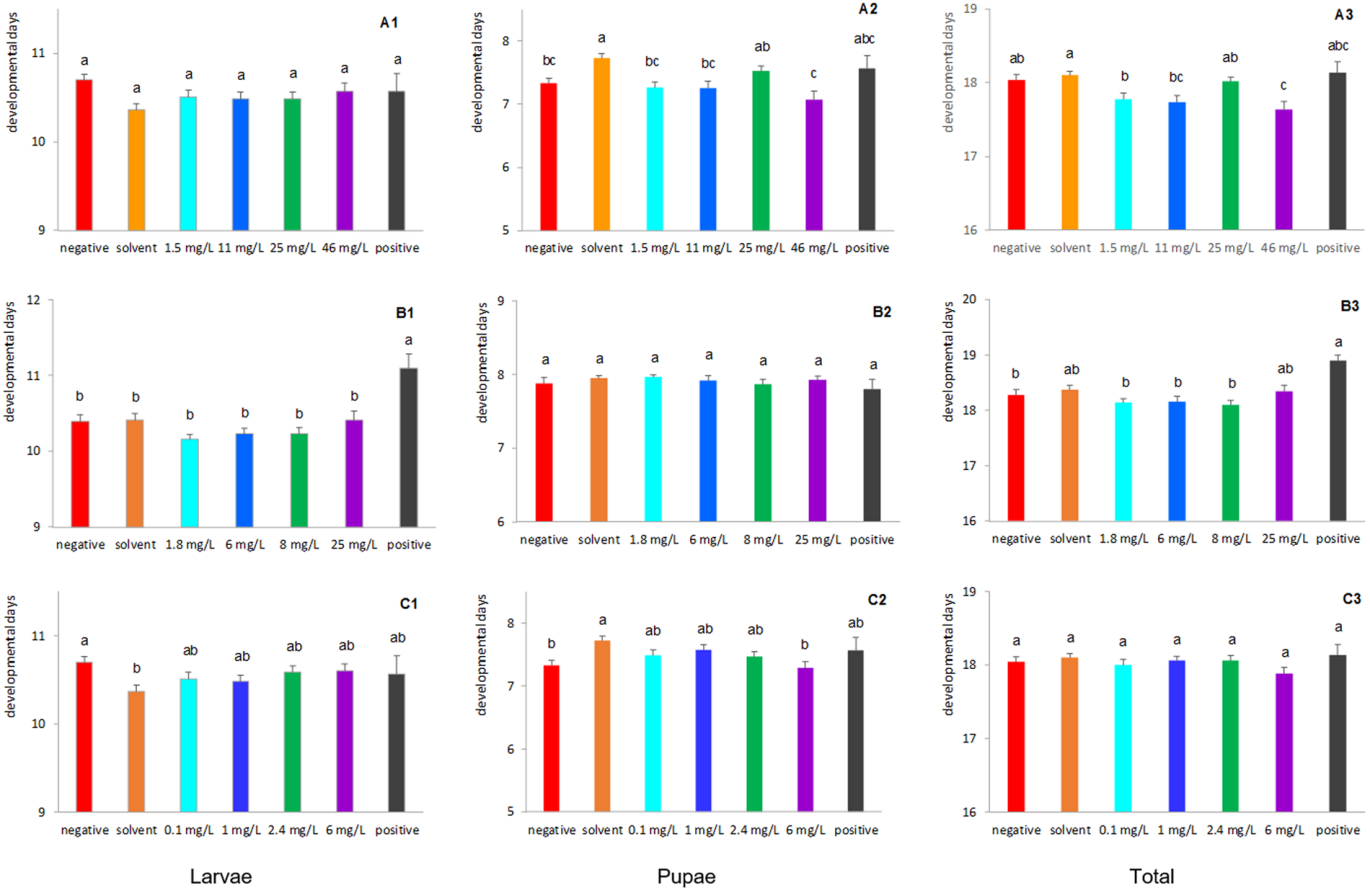
Development rate (mean days ± SE) of honey bees reared *in vitro* and exposed to (**A**) amitraz, (**B**) coumaphos or (**C**) fluvalinate in the diet on D3 thru D6 after grafting. Larvae were fed a dimethoate-contaminated diet (45 mg/L) as a positive control, acetone- or methanol-contaminated diet as a solvent control, or no contaminated diet as a negative control. Bars with the same letter within developmental rate type (larval, pupal, and total) are not different at α ≤ 0.05.

A one-way ANOVA was conducted to compare the effect of amitraz on the developmental rate of larvae (F_264_ = 2.12, *p* = 0.0515), pupae (F_264_ = 5.52, *p* < 0.0001) and both combined (total) (F_264_ = 4.83, *p = *0.0001). Exposure to amitraz did not affect the larval developmental rate (Fig. 3A1) nor pupal developmental rate in a predictable fashion (Fig. 3A2). The total developmental time was significantly lower for larvae fed a diet with 46 mg/L amitraz than for larvae fed negative and solvent control diets (Fig. 3A3).

A one-way ANOVA indicated significant differences between treatment groups in the coumaphos study for the developmental rate of larvae (F_226_ = 5.71, *p* < 0.0001), and in total (F_226_ = 3.78, *p = *0.0013), but not for pupae (F_226_ = 0.58, *p = *0.7470). Generally speaking, these differences are explained by the bees fed 46 mg/L dimethoate (positive control) in their diet which took significantly longer to develop as larvae, and then in total, than bees feeding on the negative, solvent, and treatment diets (Fig. 3B).

A one-way ANOVA was conducted to compare the effect of fluvalinate on the developmental rate of larvae (F_287_ = 2.52, *p* = 0.0218), pupae (F_287_ = 3.43, *p = *0.0028) and in total (F_287_ = 1.03, *p = *0.4094). Though significant differences between groups existed for larval and pupal development, no meaningful patterns were discernable (Fig. 3C).

### Larval weight

**Figure 4:**
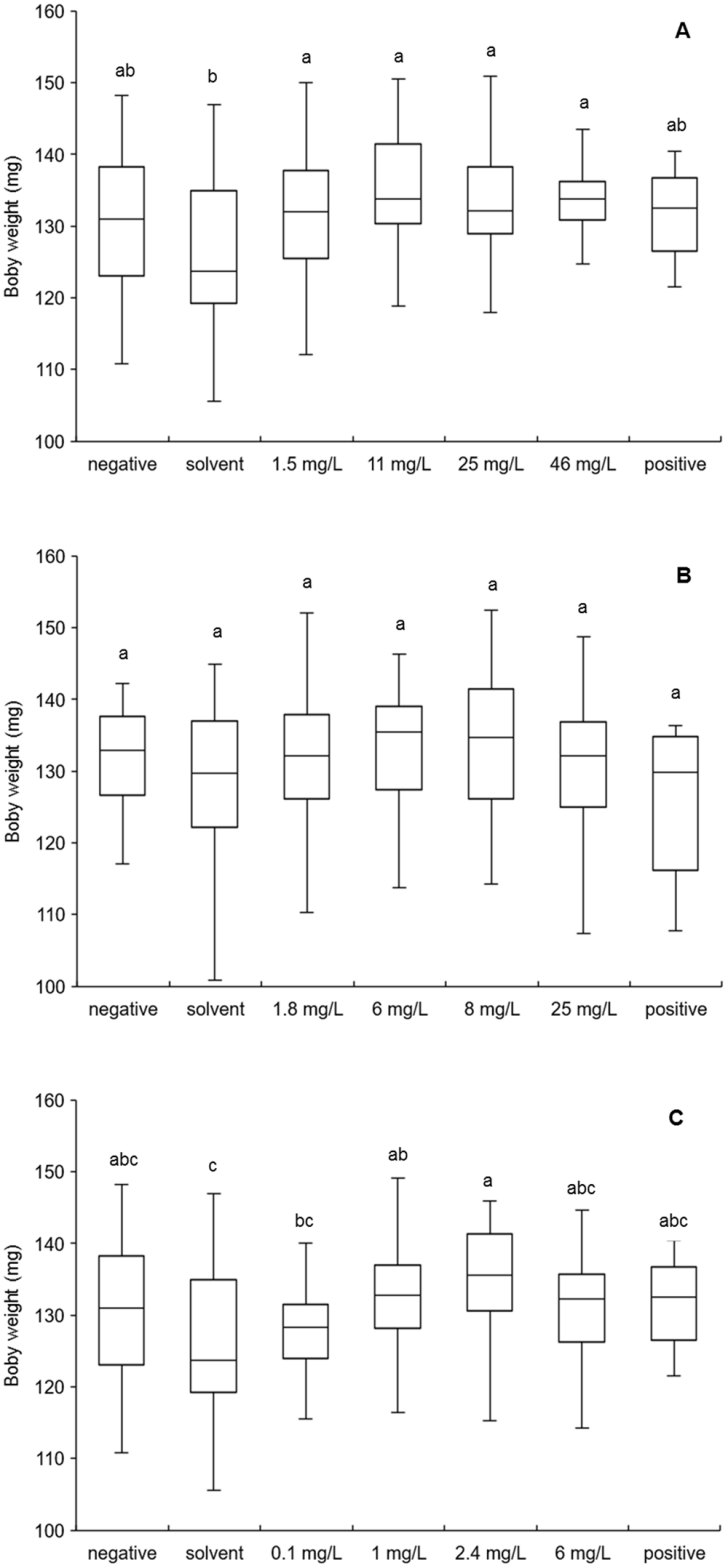
Body weight (mg) on D7 of honey bee larvae reared *in vitro* and exposed to (**A**) amitraz, (**B**) coumaphos or (**C**) fluvalinate in the diet on D3 thru D6 after grafting. Larvae were fed a dimethoate-contaminated diet (45 mg/L) as a positive control, acetone- or methanol-contaminated diet as a solvent control, or no contaminated diet as a negative control. The boxplots provide a graphical view of the median and quartiles with the error bars showing sample maximums and minimums. Bars with the same letter are not different at α ≤ 0.05.

Chronic exposure to amitraz (F_268_ = 4.39, *p* < 0.0001, Fig. 4A) and fluvalinate (F_288_ = 5.39, *p* < 0.0001, Fig. 4C) significantly affected larval weight on D7 while chronic exposure to coumaphos did not (F_225_ = 2.05, *p* = 0.06, Fig. 4B). Larvae feeding on diet containing any concentration of amitraz had significantly higher weights than did those feeding on the solvent control diet (Fig. 4A) but not than those feeding on the positive control or negative control diets. Larvae feeding on diet containing 1 mg/L and 2.4 mg/L of fluvalinate had significantly higher weights than those feeding on the solvent control diet (Fig. 4C). Larval weight was statistically similar for larvae feeding on diet with any concentration of fluvalinate, the positive control diet, and the negative control diet (Fig. 4C).

## Discussion

Honey bee larvae can be exposed to acaricides used to control *Varroa* in the hive. Amitraz, coumaphos, and fluvalinate residues have been found in pollen^7^, honey^28^, and wax^7^. We are not aware of any studies reporting pesticide residues in the glandular secretions that compose brood food or royal jelly, though such residues may be present in these worker bee secretions. However, nurse bees incorporate bee bread (a pollen product) and honey into the diet of 3+ day old larvae^29^. Thus, the larvae may be exposed to these compounds orally given their diets contain pollen and honey. They may also be exposed to the compounds via the contaminated wax in which they develop, though this potential route of exposure needs to be explored further. Dai *et al*.^30^ showed a 72 h acute LC50 for amitraz in larval food of 461.4 mg/L, coumaphos −73.5 mg/L, and fluvalinate −24.0 mg/L. Based on these data^30^ and the known residue levels of these compounds in pollen and/or honey^7,28^, we investigated the chronic toxicity of amitraz, coumaphos and fluvalinate to honey bee larvae reared *in vitro*.

Amitraz has a low acute toxicity to honey bee larvae (LC50 461.4 mg/L)^30^, but acute toxicity tests alone are not enough to assess the risk of amitraz to developing honey bees. We observed that amitraz had a low toxicity to honey bee larvae exposed to the compound chronically for four days (D3–D6) *in vitro*. Even at 1.5 mg/L (1.4× and 2.5× maximum residue in pollen and honey/nectar respectively), 11 mg/L (10× and 18 × maximum residue in pollen and honey/nectar respectively) and 25 mg/L (LC5), amitraz did not affect the survival of honey bee larvae. Only at 46 mg/L (1/10^th^ the LC50) did amitraz decrease survival when compared to that of the negative and solvent control. This concentration is much higher than the maximum residues of the amitraz breakdown product 2,4-dimethylphenyl formamide (DMPF) found in pollen (1.1 mg/L)^7^ and honey (0.6 mg/L)^28^.

In one study, larvae reared on a diet containing 8 mg/L (1.3× and 4× the maximum levels found in pollen and honey/nectar respectively) coumaphos were more likely to die during development than were control larvae^31^. In a second study, brood (3-day) survivorship was significantly higher in non-treated controls than in colonies receiving coumaphos^18^. Finally, an improvement in the survival rate of brood occurred when coumaphos residues in beeswax decreased^14^. In our experiment, 1.8 mg/L (10× and 16× mean residue in pollen and honey/nectar respectively), 6 mg/L (maximum residue in pollen) and 8 mg/L (1/10^th^ the LC50, LC5) of coumaphos in the larval diet did not affect the survival of developing honey bees. Only at 25 mg/L (1/5^th^ the LC50) did coumaphos decrease larval survival. However, this concentration is 4× higher than the maximum residue level found in pollen (6 mg/L)^7^, 12× higher than the maximum residue in honey/nectar (2 mg/L)^28^, and 60× higher than the maximum residue in royal jelly (0.4 mg/L)^32^ suggesting this compound poses a minimal risk to developing bees.

Fluvalinate residues as high as 2.7 μg/g and 0.8 μg/g have been found in pollen^7^ and honey/nectar respectively^28^. Zhu *et al*.^31^ observed that survival was impacted during development (D1–D6) for larvae reared on a diet contaminated with 3 mg/L fluvalinate. In another study, brood survival was significantly higher in non-treated controls than in fluvalinate-treated colonies^18^. We found the opposite that larvae exposed to field-relevant concentrations of fluvalinate for four days (D3–D6) did not experience reduced survival. Fluvalinate at 0.1 mg/L (mean residue in pollen and 6× mean residue in honey/nectar), 1 mg/L (10× and 60× mean residue in pollen and honey/nectar respectively) and 2.4 mg/L (1/10^th^ the LC50, maximum residue in pollen and 3× maximum residue in honey/nectar) did not affect the survival of developing honey bees. Only at 6 mg/L (LC5, 2× and 8× maximum residue in pollen and honey/nectar respectively) did fluvalinate significantly decrease the survival of larvae when compared with that of the negative control (but not the solvent control). This concentration is higher than the maximum residues found in pollen (2.7 mg/L)^7^ or honey (0.8 mg/L)^28^.

We observed that amitraz, coumaphos and fluvalinate did not impact the weight and developmental rate of the bees meaningfully. Larval and total survival were always significantly lower for individuals fed the positive control diet than for ones fed any other diet for all three compounds. This effect was not seen for pupal survival. It would be interesting to note that the survival for larvae was generally lower than that for pupae. This suggests that most of the bee death due to pesticide exposure happens in the larval stage. The effect was negligible in the pupal stage, likely with the compromised individuals already dying as larvae.

Herein, we demonstrate the chronic toxicity of amitraz, coumaphos and fluvalinate to honey bee brood. Neither appears to impact brood survival significantly at field-relevant residue levels in pollen and honey/nectar, possibly suggesting bee development of resistance to these miticides given their use in honey bee colonies for decades. Our tests represent a likely worse-case scenario exposure of larvae to these compounds given that our tests centered around residue levels seen in honey/nectar and pollen which compose only a fraction of the total volume of brood food. In our study, we took these residue levels and extrapolated them to the entire diet, rather than only the fraction composed of pollen and/or nectar, thus exaggerating any potential effect. There is little information in the literature about the residue levels of pesticides in brood food in general. However, it seems unlikely that the acaricide levels found in brood food will approach the maximum residues found in pollen or nectar/honey under normal environmental conditions, thus suggesting a negligible impact of these compounds on brood. In conclusion, we suggest that residues of amitraz, fluvalinate, and coumaphos at the levels we tested are unlikely to impact immature worker bee survival in the field, though our data do not preclude any sublethal effects that may result from bee exposure to these compounds, effects that are absent in the brood but that appear later in adult bees^33^, or possible synergisms of the compounds when they co-occur in bee colonies.

## Methods

### Acaricides

All test substances were purchased from Chem Service, Inc. (West Chester, PA 19380, United States). The name, product number, purity, and expiration of each test substance was as follows: (1) Amitraz: N-11068-250MG, purity 98.7%, expiration 6/30/2018; (2) coumaphos: N-11507-100MG, purity 99.3%, expiration 10/31/2019; (3) fluvalinate: N-13263-100MG, purity 95.0%, expiration 10/31/2020.

### Honey bee rearing conditions

Experiments were conducted at the Honey Bee Research and Extension Laboratory, Entomology and Nematology Department, University of Florida (Gainesville, FL, USA) during May – July 2016. Honey bee larvae were reared *in vitro* according to Schmehl *et al*.^26^. During the rearing process, the larvae were fed increasing volumes of three diet compositions (A, B, and C). The larval diets were composed as follows: Diet A - royal jelly 44.25%, glucose 5.3%, fructose 5.3%, yeast extract 0.9% and water 44.25%; Diet B - royal jelly 42.95%, glucose 6.4%, fructose 6.4%, yeast extract 1.3% and water 42.95%; Diet C - royal jelly 50%, glucose 9%, fructose 9%, yeast extract 2% and water 30%^26^.

Five honey bee queens were caged on empty wax combs within their respective colonies. The queens were caged for 24 hours, during which time they laid eggs in the combs. After 24 hours, the queens were released, and the combs of eggs were re-caged (to prevent the queen from laying more eggs in the comb) and returned to the hive to allow the eggs to hatch. Following this, the combs containing the resulting larvae [age time t = 87 ± 12 h (75 h after the queens were released)] were transported to the laboratory for grafting. At the laboratory, larvae were transferred from the comb to sterile, 48-well tissue culture plates (STCPs) that had been prepared with 20 μL of diet A in each cell cup (Day D1). The STCPs were placed horizontally in a larval growth chamber maintained at 94% R.H. and 35 °C. On day 3, each larva was fed 20 μL of diet B. On D4, 5 and 6, each larva was fed 30 μL, 40 μL, and 50 μL, respectively, of diet C. Larvae were transferred individually from the larval STCPs to pupal STCPs when all available diet had been consumed (as early as day 7). Pupal STCPs were maintained horizontally in a pupal growth chamber maintained at 75% R.H. and 35 °C. Adult worker bees began to eclose as soon as 18 days after grafting.

### Experimental design

Four concentrations of each test substance were selected based on previously published acutely toxic^30^ and field relevant doses^7^. The concentrations tested for each substance were chosen based on available toxicology data and pollen residue data, the latter instead of nectar/honey residue data given that all three compounds have been found at higher residue levels in pollen than in nectar/honey^7,28^. The test concentrations were as follows: amitraz: 1.5 mg/L (10× mean residue level reported in pollen)^7^, 11 mg/L (10× maximum residue level reported in pollen)^7^, 25 mg/L (LC5)^30^ and 46 mg/L (1/10^th^ the LC50)^30^; coumaphos: 1.8 mg/L (10× mean residue level reported in pollen)^7^, 6 mg/L (maximum residue level reported in pollen)^7^, 8 mg/L (1/10^th^ the LC50, LC5)^30^ and 25 mg/L (1/5^th^ the LC50)^30^; fluvalinate: 0.1 mg/L (mean residue level reported in pollen)^7^, 1 mg/L (10× mean residue level reported in pollen)^7^, 2.4 mg/L (either 1/10^th^ the LC50^30^; or maximum residue level reported in pollen^7^) and 6 mg/L (LC5)^30^. Amitraz and fluvalinate were dissolved in methanol to prepare stock solution and coumaphos was dissolved in acetone. The solvent accounted for 0.5% of the volume in final diets.

The following treatments were conducted for each test solution: four concentrations of the test solution, negative control, solvent control, and 45 mg/L dimethoate (positive control). Five replicates were conducted for each treatment. Larvae tested within each replicate were sourced from a single colony and each replicate was sourced from a different colony.

A surplus of larvae was grafted for each replicate. On D3, a minimum of twelve robust larvae were selected for each treatment group and fed 20 μL of diet B containing the test solution appropriate to the group’s assigned treatment. On D4, 5, and 6, the larvae were fed 30, 40, and 50 μL, respectively, of diet C containing the appropriate test solution.

### Endpoints

Larval survival was assessed each day by viewing larvae under a dissecting microscope at which time spiracular movement (opening/closing) was noted. The individual was considered dead if no spiracular movement was detected. Pupal survival was monitored daily by visual inspection of the pupae. Dead prepupae and pupae were recognized by occasional black or white sub-dermal necrotic stains or visible wilting. Any larvae or pupae determined to be dead were removed from the STCPs. Larval, pupal, and total survival rates were calculated for each treatment group using the following formulas.

The survival rate (% survival or “survival”) was calculated for each treatment group as:

$$\begin{array}{c}\begin{array}{l}{\rm{larval}}\,{\rm{survival}}=(\#\,{\rm{larvae}}\,{\rm{that}}\,{\rm{reached}}\,\mathrm{D10}/\#\,{\rm{larvae}}\,{\rm{grafted}})\times 100\\ {\rm{pupal}}\,{\rm{survival}}=(\#\,{\rm{adults}}\,{\rm{that}}\,\mathrm{eclosed}/\#\,{\rm{larvae}}\,{\rm{that}}\,{\rm{reached}}\,{\rm{D10}})\times 100\end{array}\\ {\rm{Total}}\,{\rm{survival}}=(\#\,{\rm{adults}}\,{\rm{that}}\,\mathrm{eclosed}/\#\,{\rm{larvae}}\,{\rm{grafted}})\times 100.\end{array}$$

Additionally, the developmental rate was calculated for each treatment group as:

$$\begin{array}{l}{\rm{Larval}}\,{\rm{developmental}}\,{\rm{rate}}=({\rm{date}}\,{\rm{of}}\,{\rm{pupation}}\,{\rm{initiation}}\,-\,{\rm{grafting}}\,{\rm{date}}+1)\\ {\rm{Pupal}}\,{\rm{developmental}}\,{\rm{rate}}=({\rm{emergence}}\,{\rm{date}}\,-\,{\rm{date}}\,{\rm{of}}\,{\rm{pupation}}\,{\rm{initiation}})\\ {\rm{Total}}\,{\rm{developmental}}\,{\rm{rate}}=({\rm{emergence}}\,{\rm{date}}\,-\,{\rm{grafting}}\,{\rm{larva}}\,{\rm{date}}+1).\end{array}$$

Larvae were grafted at 24 h, so we added “ + 1” for calculating larval developmental rate and total developmental rate.

Finally, larval fresh body weight at D7 was calculated for each larva immediately prior to transfer to the pupal STCP. Fresh body weight was calculated by weighing the weight of the larval cell cup and mature larva. The larva was then transferred to the pupal STCP, and the empty larval cell cup was weighed again. The weight of each larva was calculated as follows: larval weight at D7 = weight of larval cell cup with larva − weight of empty larval cell cup.

### Statistical analysis

Statistical analyses were performed using the SAS 9.2 software program (USA)^34^. The survival rate data (survival over time) were tested with a Kaplan-Meier analysis. Developmental rate data were normalized by arcsine-square root transformation of the proportions to allow for comparisons between groups and the controls via ANOVA and Tukey’s HSD tests. Furthermore, ANOVAs and Tukey’s HSD tests were used to compare larval weight at D7 and % survival (“survival”) among the experimental groups.

## Electronic supplementary material

Supplementary Information
