# Supplementary material for: Chronic toxicity of amitraz, coumaphos and fluvalinate to Apis mellifera L. larvae reared in vitro
Source: Sci Rep. 2018 Apr 4;8:5635. doi: 10.1038/s41598-018-24045-3 (PMC5884784; doi:10.1038/s41598-018-24045-3)

Chronic toxicity of amitraz, coumaphos and fluvalinate to *Apis mellifera* L. larvae reared *in vitro*

Pingli Dai[1,2]*, Cameron J. Jack[2], Ashley N. Mortensen[2], Tomas A. Bustamante[2], James D. Ellis[2]*

1. Key Laboratory of Pollinating Insect Biology, Institute of Apicultural Research, Chinese Academy of Agricultural Sciences, Beijing 100093, China

2. Honey Bee Research and Extension Laboratory, Entomology and Nematology Department, University of Florida, Gainesville, Florida 32611, USA

*Corresponding authors. Tel. (fax): +86 10 62597285. E-mail address: daipingli@caas.cn; jdellis@ufl.edu.

Additional information

Table S1. Data analysis on survival of worker honey bees to eclosion when exposed to amitraz chronically as larvae. NC = negative control, SC = solvent control (methanol), PC = positive control (dimethoate).

Table S2. Data analysis on survival of worker honey bees to eclosion when exposed to coumaphos chronically as larvae. NC = negative control, SC = solvent control (methanol), PC = positive control (dimethoate).

Table S3. Data analysis on survival of worker honey bees to eclosion when exposed to fluvalinate chronically as larvae. NC = negative control, SC = solvent control (methanol), PC = positive control (dimethoate).

Fig. S1. Honey bees reared *in vitro* from larvae to adult emergence. D1 = the grafting day [time t = 87 ± 12 h (75 h after the queens were released)].

Fig. S2. Honey bee survival on D3 – D21 when exposed as larvae to coumaphos on D3, D4, D5, and D6. In each image, the concentration of the test compound increased in the columns from left to right (from C1 – C4: C1 = 1.8 mg/L, C2 = 6 mg/L, C3 = 8 mg/L, C4 = 25 mg/L). Replicates from up to down (R1 – R5) each constitute larvae collected from different colonies. A blank space in the tissue culture plate indicates that the developing bee formerly in the well died and was removed.

Table S1. Data analysis on survival of worker honey bees to eclosion (total survival) when exposed to amitraz chronically as larvae. NC = negative control, SC = solvent control (methanol), PC = positive control (dimethoate).

**Comparison**	**Chi-squares**	***p***
1.5 mg/L - NC	1.3772	0.2406
1.5 mg/L - SC	0.5447	0.4605
1.5 mg/L - PC	66.2907	<.0001
11 mg/L - NC	9.8291	0.0017
11 mg/L - SC	2.2017	0.1379
11 mg/L - PC	43.0856	<.0001
25 mg/L - NC	9.5206	0.0020
25 mg/L - SC	1.8169	0.1777
25 mg/L - PC	51.8839	<.0001
46 mg/L - NC	29.9830	<.0001
46 mg/L - SC	15.3937	<.0001
46 mg/L - PC	33.6018	<.0001

Table S2. Data analysis on survival of worker honey bees to eclosion (total survival) when exposed to coumaphos chronically as larvae. NC = negative control, SC = solvent control (acetone), PC = positive control (dimethoate).

**Comparison**	**Chi-squares**	***p***
1.8 mg/L - NC	2.1326	0.1442
1.8 mg/L - SC	1.1328	0.2872
1.8 mg/L - PC	58.2829	<.0001
6 mg/L - NC	1.5594	0.2118
6 mg/L - SC	0.8482	0.3571
6 mg/L - PC	54.3242	<.0001
8 mg/L - NC	0.1351	0.7132
8 mg/L - SC	0.0030	0.9563
8 mg/L - PC	62.4435	<.0001
25 mg/L - NC	19.5144	<.0001
25 mg/L - SC	17.0874	<.0001
25 mg/L - PC	11.8077	0.0006

Table S3. Data analysis on survival of worker honey bees to eclosion (total survival) when exposed to fluvalinate chronically as larvae. NC = negative control, SC = solvent control (methanol), PC = positive control (dimethoate).

**Comparison**	**Chi-squares**	***p***
0.1 mg/L - NC	3.8139	0.0508
0.1 mg/L - SC	0.0322	0.8576
0.1 mg/L - PC	58.9156	<.0001
1 mg/L - NC	0.8873	0.3462
1 mg/L - SC	0.8391	0.3596
1 mg/L - PC	65.1555	<.0001
2.4 mg/L - NC	2.1634	0.1413
2.4 mg/L - SC	0.1306	0.7178
2.4 mg/L - PC	63.4129	<.0001
6 mg/L - NC	11.0043	0.0009
6 mg/L - SC	2.8979	0.0887
6 mg/L - PC	44.2842	<.0001

Fig. S1. Honey bees reared *in vitro* from larvae to adult emergence. D1 = the grafting day [time t = 87 ± 12 h (75 h after the queens were released)].

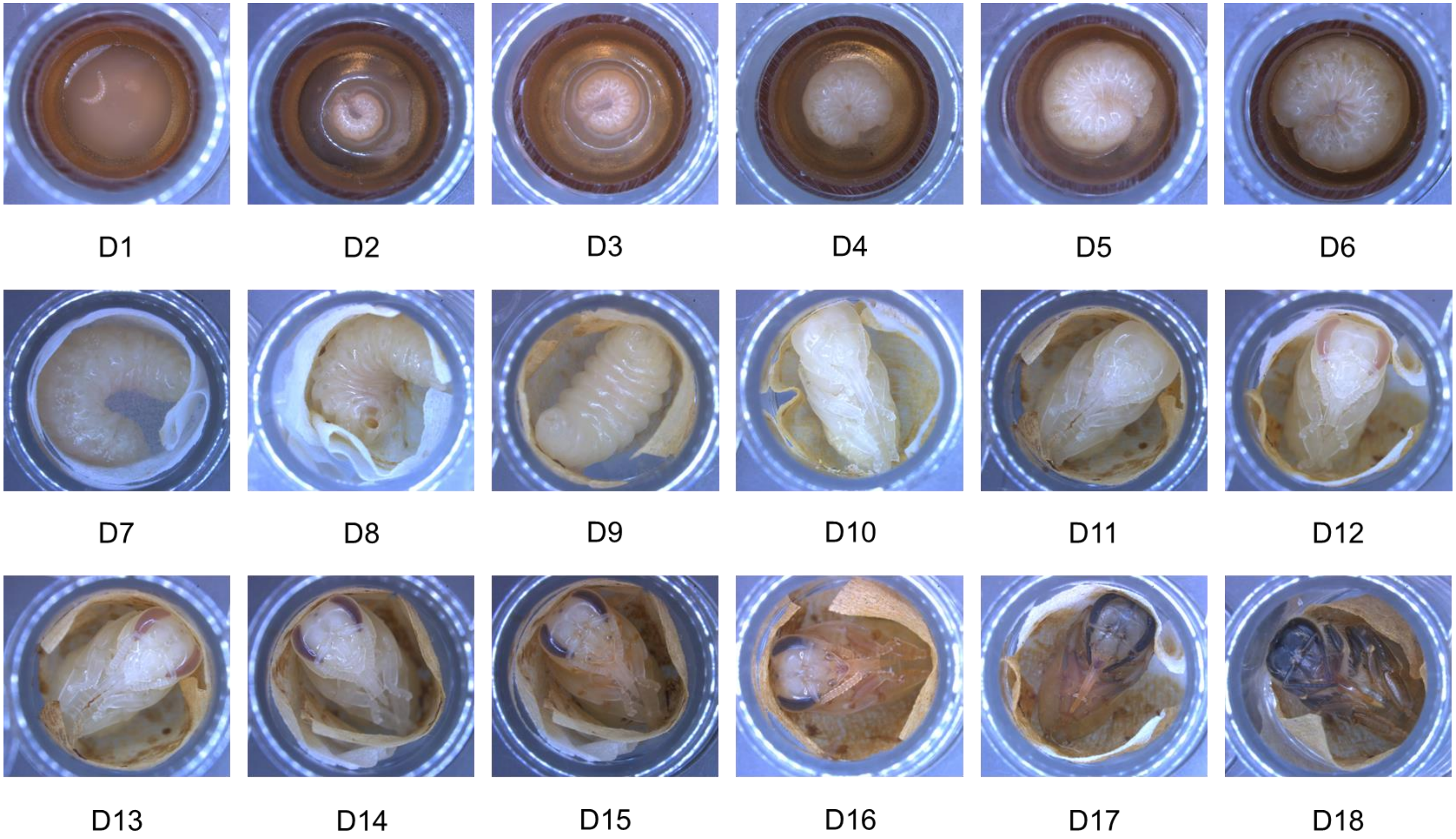

Fig. S2. Honey bee survival on D3 – D21 when exposed as larvae to coumaphos on D3, D4, D5, and D6. In each image, the concentration of the test compound increased in the columns from left to right (from C1 – C4: C1 = 1.8 mg/L, C2 = 6 mg/L, C3 = 8 mg/L, C4 = 25 mg/L). Replicates from up to down (R1 – R5) each constitute larvae collected from different colonies. A blank space in the tissue culture plate indicates that the developing bee formerly in the well died and was removed.

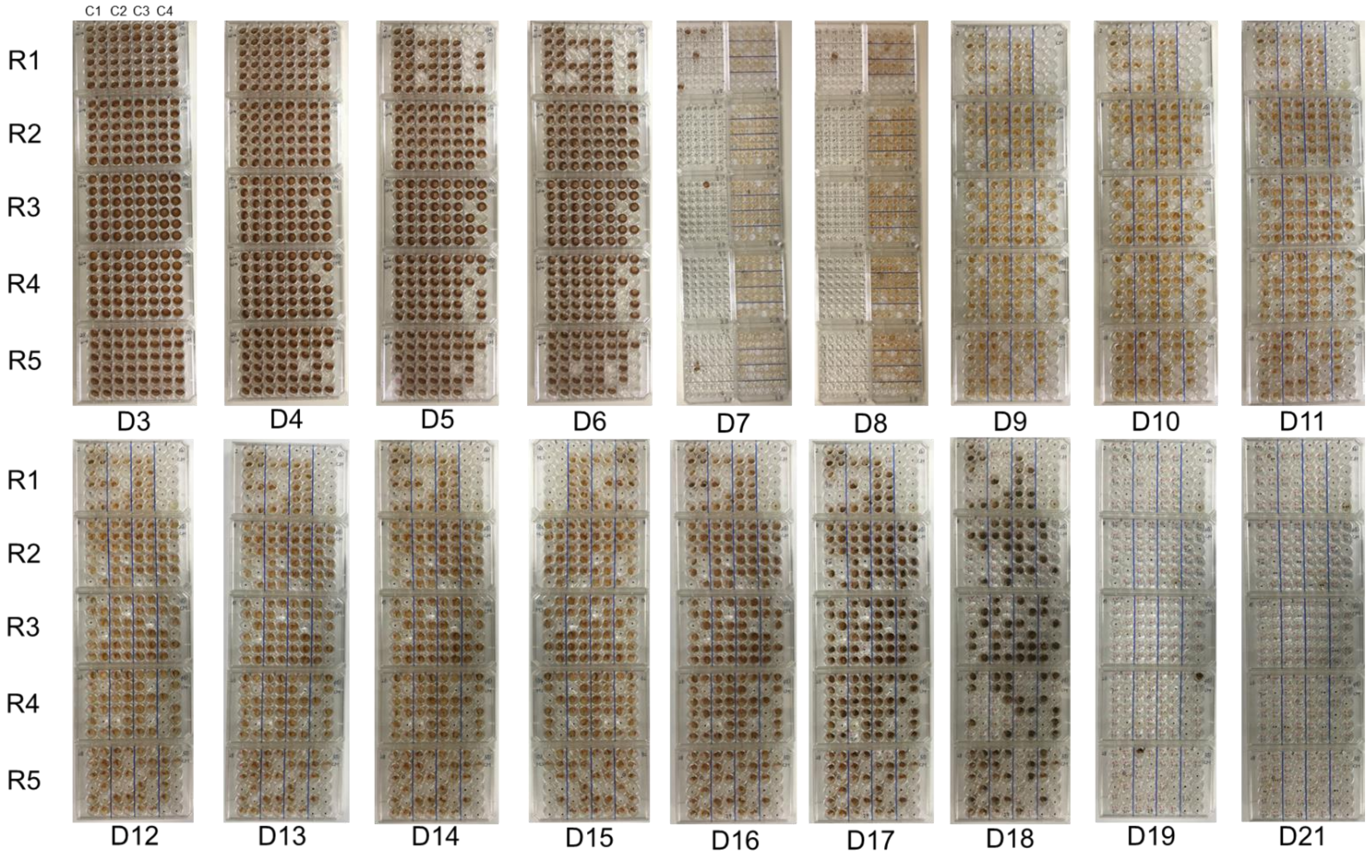

Supplement: Supplementary file 1 — Supplementary Information [file 41598_2018_24045_MOESM1_ESM.pdf]
